# Outcomes and Complications After LUMiC^®^ Endoprosthetic Reconstruction of Periacetabular Defects—A Retrospective Cohort Analysis

**DOI:** 10.3390/life16060955

**Published:** 2026-06-05

**Authors:** Adrian Su Niemann, Ricardo Ramon, Jorge Mayor, Maximilian Koblenzer, Gökmen Aktas, Tarek Omar Pacha, Sebastian Decker, Tilman Graulich

**Affiliations:** 1 Department of Trauma Surgery, Hannover Medical School, 30161 Hannover, Germany; ramonvelastegui.sebastian@mh-hannover.de (R.R.); mayorramirez.jorge@mh-hannover.de (J.M.); koblenzer.maximilian@mh-hannover.de (M.K.); aktas.goekmen@mh-hannover.de (G.A.); omarpacha.tarek@mh-hannover.de (T.O.P.); decker.sebastian@mh-hannover.de (S.D.); tilman.graulich@pius-hospital.de (T.G.); 2 Department of Orthopaedic and Trauma Surgery, Pius Hospital Carl von Ossietzky University of Oldenburg, 26219 Oldenburg, Germany

**Keywords:** LUMiC prosthesis, hemipelvic reconstruction, acetabular defect, limb-salvage surgery, periacetabular tumor, endoprosthetic reconstruction, implant survival, complication rate

## Abstract

(1) Background: The reconstruction of periacetabular defects after tumor resection remains one of the most challenging procedures in orthopaedic oncology. The modular LUMiC^®^ system was designed to improve fixation stability and reduce implant-related complications compared with earlier hemipelvic prostheses. We investigated patient and implant survival after LUMiC^®^ reconstruction, complication types and functional outcomes. (2) Methods: Eighteen patients (8 men, 10 women; mean age 58.9 years) underwent LUMiC^®^ endoprosthetic reconstruction between 2011 and 2025. Kaplan–Meier analysis was used to estimate patient and implant survival. Complications were categorized according to Henderson. Functional results were evaluated at follow-up using MSTS and TESS. (3) Results: Mean follow-up was 35.52 months (SD 36.89). Overall implant survival was 72.2%. Instability (27.8%) and infection (16.7%) were the leading complications. Two-thirds of patients required at least one revision (mean 3.1 revisions per case). Metastatic disease reduced patient survival (*p* = 0.012) but did not affect implant longevity (*p* = 0.31). Functional outcomes were available for only 3 of 18 patients and should therefore be regarded as exploratory. Mean MSTS was 58.9% (SD 21.43) and mean TESS was 73.4% (SD 6.836). (4) Conclusions: Despite high revision rates, the LUMiC^®^ prosthesis provides durable fixation. Early revision does not appear to compromise implant survival. However, the small and heterogeneous cohort represents a limiting factor of this study.

## 1. Introduction

Internal hemipelvectomy is an important limb-salvage procedure in the treatment of malignant bone tumors and soft-tissue neoplasm of the pelvic region [1]. The procedure is technically highly demanding because of the complexity of pelvic anatomy, including the presence of major neurovascular structures and the substantial osseous and soft-tissue defects that remain following resection [2,3]. One of the major challenges after extensive periacetabular resection is the loss of the hip joint, resulting in mechanical discontinuity between the pelvic girdle and the lower extremity [4].

A well-established system for describing the extent of pelvic resection is the Enneking classification. The Enneking classification consists of four resection levels [5]. Type P1 refers to resection of the ilium above the acetabulum, whereas type P2 involves the resection of the acetabulum. Type 3 corresponds to the removal of the ischiopubic region below the acetabulum, and type P4 refers to the resection of the sacrum. Following pelvic bone resection, a substantial bone defect remains, necessitating a reconstructive treatment.

Over time, various endoprosthesis models have been developed. In 1990, Nieder et al. reported on a saddle prosthesis (Waldemar Link GmbH, Hamburg, Germany) for reconstruction after resection of the acetabulum [6]. A U-shaped saddle is mounted onto the femoral stem of a hip arthroplasty. Provided that sufficient bone stock remains in the ilium, the residual iliac wing can rest onto the saddle component, thereby eliminating the need for an acetabular implant. However, complications such as the destruction of the iliac wing and cranial migration of the prosthesis remain challenging [7]. In addition, the saddle prosthesis has poor long-term function due to limited hip flexion [8].

To tackle these complications, a coned hemi-pelvic prosthesis was introduced in 2003 (Stanmore Worldwide Ltd., Elstree, UK) [9]. Because the prosthesis is anchored to the pelvis using a conical, fluted hydroxyapatite-coated stem, even a minimum amount of residual ilium is sufficient to provide stability. While the coned hemi-prosthesis, also known as the ice-cream cone prosthesis, shines with simplicity and easy implantation, Hu et al. demonstrated in their biomechanical study that high-stress concentrations occur at the fixation site, which may contribute to postoperative loosening [10]. Therefore, Hu et al. favored 3D-printed hemipelvic prostheses for the restoration of the pelvic ring, owing to their more physiological stress transmission. Regarding the clinical outcome, a poor functional Musculoskeletal Tumor Society Score (MSTS) of 38% for saddle prostheses [11] has been reported, whereas custom-made hemipelvic prostheses reached a Musculoskeletal Tumor Society Score of 72% [7].

A shared challenge across all types of hemipelvic prosthesis is the high risk of infection and dislocation [12,13,14]. For pelvic reconstruction, the use of an allograft represents an alternative to a prosthesis [15]. In comparison to hemipelvic prosthesis, however, pelvic allografts are linked to an increased incidence of complications [16]. In particular, nonunion and allograft fractures continue to pose a challenge [17].

Another alternative is hip transposition. Hip transposition is a procedure in which the femoral head is fixed to the remaining bone after periacetabular resection using bone anchors or transosseous sutures, often in combination with a mesh [18]. Since no prosthesis needs to be implanted in hip transposition, lower complication rates have been reported [19]. However, leg length discrepancy frequently occurs after hip transposition [20], as well as a prolonged recovery period [21]. With regard to functionality, an MSTS score from 45% to 62% has been reported after hip transposition [21,22]. So far, there is no clear consensus regarding the reconstruction of periacetabular defects following tumor resection.

Introduced in 2008, the LUMiC^®^ prosthesis (Implantcast, Buxtehude, Germany) was designed to minimize implant-associated complications [23]. The LUMiC^®^ prosthesis is a modular device consisting of a stem and an acetabular cup connected to the stem with an option for rotational adjustment due to sawteeth at the junction [24]. The aim of this study was to analyze the outcomes of the LUMiC^®^ prosthesis in our heterogeneous patient cohort.

## 2. Materials and Methods

We retrospectively analysed all patients aged over 18 years who received a LUMiC^®^ prosthesis (Implantcast, Buxtehude, Germany) between 2011 and 2025 at our level-1 trauma and sarcoma center. Inclusion criteria were the implantation of LUMiC prosthesis and the availability of digital data. The exclusion criterion was an age under 18 years at the time of surgery. General patient data was gathered from patient records and listed using Microsoft Excel 2019 MSO (Version 2408 Build 16.0.17298.20114, Microsoft Corporation, Redmond, WA, USA).

The operative procedure was performed according to the indication and tumor extension under general anesthesia in lateral positioning, most commonly via a posterior Kocher–Langenbeck approach or, when appropriate, an extended iliofemoral approach according to Smith Peterson. Preoperative digital planning was performed using mediCAD (Version 7.0, mediCAD Hectec GmbH, Landshut, Germany) to optimize the reconstruction of the hip center and the leg length. Routine postoperative care included, when indicated, intensive care unit monitoring followed by ward-based care until satisfactory wound healing and adequate mobilization were achieved. Postoperative follow-up was conducted according to tumor-specific guidelines, at least annually, and in non-oncological cases at 6 weeks, 3 months, 6 months, 12 months and yearly thereafter. The last documented outpatient visit was defined as final unless death or amputation had been recorded earlier.

Complications were divided into groups according to the classification described by Henderson [25]. Briefly, the Henderson classification categorizes complications following joint arthroplasty into five main types: Type I (soft tissue failure such as instability or wound problems), Type II (aseptic loosening), Type III (the structural failure of the implant or bone), Type IV (infection), and Type V (tumor progression or local recurrence, mainly in tumor prostheses). If a complication according to Henderson occurred, the patients were classified according to the first complication that appeared. If a second complication according to Henderson occurred during the course after the initial manifestation of a Henderson complication, the second complication was also recorded. The retrospective classification of complications in all patients was performed by a specialist physician at the clinic.

For functional evaluation, the MSTS and the Toronto Extremity Salvage Score (TESS) were used during follow-up. Both scores are functional outcome measures used to evaluate patients after limb-salvage surgery or amputation for musculoskeletal tumors. The MSTS score is clinician-reported and assesses pain, function, emotional acceptance, use of supports, walking ability, and gait (for lower limbs) or hand positioning, dexterity, and lifting ability (for upper limbs), giving a total score out of 30. The TESS score is patient-reported and focuses on daily activities such as mobility, self-care, and social function. High values indicate good functional outcome and quality of life.

While wound healing after total hip arthroplasty is generally completed within 14 days [26], a cutoff of 21 days was pre-specified in the present study to define “early revision” in order to account for the greater surgical extent of hemipelvic reconstruction. Since hemipelvectomy with hemipelvic reconstruction represents a substantially more invasive and biologically complex procedure than conventional total hip arthroplasty, prolonged wound healing times can be expected. The 21-day threshold was used as an arbitrary cutoff and was not based on a scientifically established duration of wound healing after hemipelvic reconstruction. To date, we are not aware of any study that has systematically evaluated the time to complete wound healing following hemipelvectomy. In the study by Chao et al., an arbitrary dichotomization between early and late revision was defined using a 30-day threshold [27].

Statistical analysis was performed with GraphPad Prism (Version 11.0.2, GraphPad Software, Boston, MA, USA). Categorical variables were presented as number and percentage. Values were tested for normal distribution. One data point identified as an outlier based on the 1.5 × IQR rule was excluded from the box plot for visual clarity. Statistical significance was considered with a two-tailed *p*-value of *p* < 0.05. Survival analysis was performed using Kaplan–Meier curves, and differences between groups were assessed using the log-rank test. Patient survival was assessed using all-cause mortality. For implant survival, the endpoint was the complete explantation of the LUMiC^®^ prosthesis.

Each author certifies that all investigations were conducted in conformity with ethical principles of research. Ethical approval was given through the Institutional Review Board (Number: 11916-BO-K-2025).

## 3. Results

Overall, 18 patients underwent surgery with an LUMIC^®^ prosthesis at our institution between 2011 and 2025. Of these patients, seven died, and one patient underwent amputation due to postoperative infection. The mean follow up was 35.52 months (SD 36.89). No differences in tumor localization could be identified.

Of the 18 patients, 44.44% were male and 55.56% were female. The mean age was 58.92 years (SD 19.23 years). The most common indication for surgery and the implantation of LUMiC^®^ prosthesis was a bone tumor (*n* = 9), with chondrosarcoma being the most frequent entity (Table 1).

The majority of patients were diagnosed with high-grade tumors, and metastases were present in 31.25% of cases (Table 2).

The most frequent tumor size category according to the TNM classification was T2. In two cases, surgery was performed due to prosthetic failure and periprosthetic fracture with a consecutive acetabular bony defect. A total of 22.22% of patients had undergone prior pelvic and/or femoral surgery.

The most frequently performed resection level according to Enneking classification was a combined P2 and P3 resection (Table 3). The mean duration of surgery was 441 min (SD 194.5 min), and the mean postoperative hospital stay was 38.78 days (SD 25.71 days).

Cup size 54 was the most frequently uncemented LUMiC^®^ prosthesis. In over half of the cases, a dual-mobility cup was used (Table 4). The most frequently used LUMiC stem size was 8 × 85 (*n* = 10). The Ecofit 17.5 (*n* = 3) and MUTARS 14 × 120 (*n* = 3) were the most commonly implanted femoral stems.

**Figure 1:**
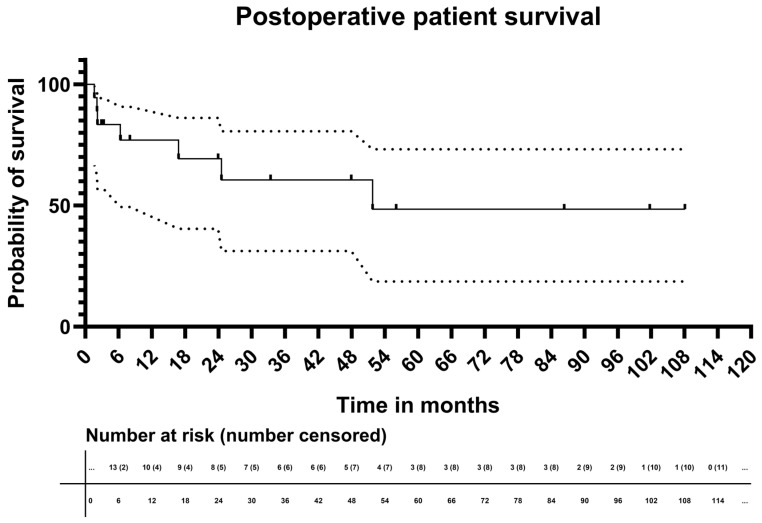
Kaplan–Meier analysis of overall survival following LUMiC^®^ prosthesis implantation. Mean survival time was 51.78 months. Survival probability is shown over time in months. Tick marks indicate censored observations. The number of patients at risk is displayed below the *x*-axis. The event was defined as death from any cause. Patients without an event at last follow-up were censored at that time point. Dotted lines represent 95% confidence interval.

Kaplan–Meier curve analysis showed that mean patient survival time was 51.78 months (see Figure 1).

**Figure 2:**
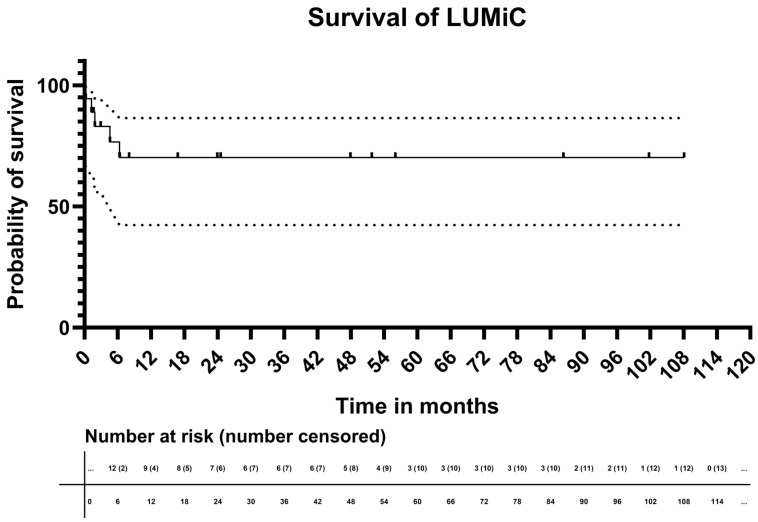
Kaplan–Meier curve survival analysis for LUMiC^®^ prosthesis. Overall survival was 72.22%. Survival probability is shown over time in months. Tick marks indicate censored observations. The number of patients at risk is displayed below the *x*-axis. The event was defined as complete explantation of the LUMiC^®^ prosthesis. Patients without an event at last follow-up were censored at that time point. Dotted lines represent 95% confidence interval.

A subgroup analysis was conducted between patients diagnosed with chondrosarcoma, osteosarcoma, other oncological diagnoses and non-oncological diagnoses (*p* = 0.5225) (see Appendix A). Given the very small sample sizes in some subgroups, particularly the osteosarcoma and non-oncological cohorts, these exploratory findings should be interpreted with caution, as some subgroup curves are based on extremely small patient numbers. In our cohort, the survival rate of LUMiC^®^ prostheses was 72.22% (*n* = 13) (Figure 2). A subgroup analysis was conducted between patients diagnosed with chondrosarcoma, osteosarcoma, other oncological diagnoses and non-oncological diagnoses (*p* = 0.5663) (see Appendix B). Given the very small sample sizes in some subgroups, particularly the osteosarcoma and non-oncological cohorts, these exploratory findings should be interpreted with caution, as some subgroup curves are based on extremely small patient numbers.

**Figure 3:**
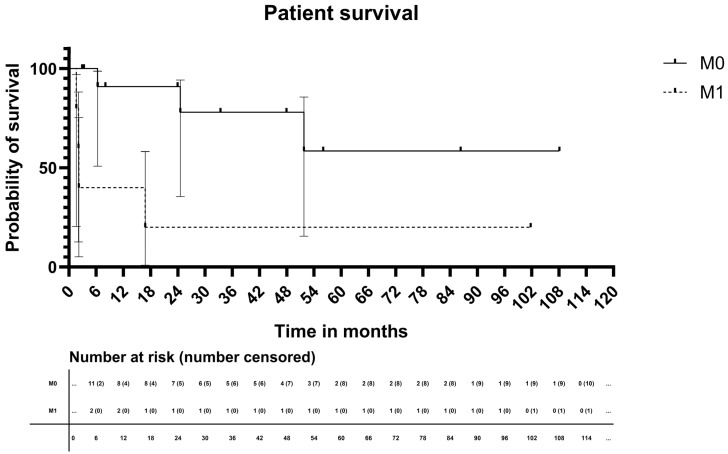
Kaplan–Meier analysis of overall survival following LUMiC^®^ prosthesis implantation. Survival curves for patients without metastasis at time of surgery (M0) and with metastasis (M1) are shown. The difference between groups was evaluated using the log-rank test (*p* = 0.0122). Survival probability is shown over time in months. Tick marks indicate censored observations. The number of patients at risk is displayed below the *x*-axis. The event was defined as death from any cause. Patients without an event at last follow-up were censored at that time point. Bars represent 95% confidence interval.

A significantly lower survival rate in patients with metastases was found in comparison to patients without metastases (*p* = 0.0122) (see Figure 3). Due to the limited number of events and the resulting lack of statistical power, no firm conclusion regarding differences in patient survival can be drawn from these data. In particular, the subgroup of patients with metastases comprised an extremely limited number of cases, and the corresponding findings should therefore be interpreted with considerable caution.

**Figure 4:**
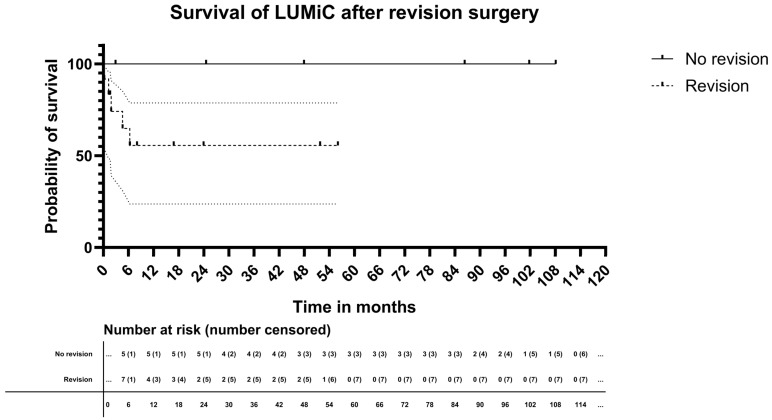
Kaplan–Meier survival analysis for LUMiC^®^ prostheses in dependence on revision surgery. Survival curves for LUMiC prostheses undergoing revision surgery and no revision surgery are shown. The difference between groups was evaluated using the log-rank test (*p* = 0.0810). Survival probability is shown over time in months. Tick marks indicate censored observations. The number of patients at risk is displayed below the *x*-axis. The event was defined as complete explantation of the LUMiC^®^ prosthesis. Patients without an event at last follow-up were censored at that time point. Dotted lines represent 95% confidence interval.

Taking a closer look at the time until the first revision surgery, revision after 21 days did not lead to a decreased survival rate of LUMiC^®^ prostheses in comparison to revision within 21 days. The survival rates of LUMiC prostheses in patients who did not require a revision procedure did not show any difference in comparison to patients who underwent a revision procedure (Figure 4). Given the limited number of events and the resulting lack of statistical power, this subgroup comparison is exploratory and should be interpreted with caution. Furthermore, both subgroups comprised a limited number of cases, which restricts the interpretability of the subgroup analyses.

Two-thirds of the patients needed revision surgery. In cases of revision procedure, the mean number of revision surgery was 3.16. The median time until the first revision was 17 days (interquartile range 23). Mean time until first revision was 21.55 days (SD 12.84). Instability (Henderson 1A) was the main complication leading to revision, whereas early periprosthetic infection was the main secondary complication (Table 5).

At final follow-up, three questionnaires were completely returned for final evaluation. The mean MSTS was 58.89 and mean TESS was 73.37 (Table 6). The first patient was a 33-year-old man with a peripheral nerve sheath tumor who did not require any revision surgeries. The second patient was a 60-year-old woman with a solitary fibrous tumor and no revision surgeries. The third patient was a 30-year-old woman with osteosarcoma who underwent two revision surgeries.

## 4. Discussion

The reconstruction of periacetabular bone defects remains one of the most technically demanding procedures in orthopaedic oncology. The introduction of modular hemipelvic prostheses such as the LUMiC^®^ system was intended to overcome the limitations of earlier designs, including saddle and “ice-cream-cone” prostheses, which were prone to mechanical failure and a restricted range of motion. Our 13-year single-center experience provides long-term data on implant survival, complication patterns, and functional recovery after LUMiC^®^ reconstruction.

The modular LUMiC^®^ endoprosthesis was developed to provide more reliable fixation and improved load transfer. The implant features a conical, self-impacting stem of variable diameter that achieves secure press-fit fixation within the residual posterior ilium, thereby enhancing resistance to aseptic loosening. The acetabular component can accept either a large femoral head or a dual-mobility articulation to reduce the risk of postoperative instability. The modular junction between stem and cup allows precise rotational adjustment, enabling the correction of version and optimization of joint stability. Because the system uses a standardized surgical technique and a modular configuration, it can be adapted to diverse anatomical situations and resection extents, provided that sufficient iliac bone is preserved. This modularity also permits timely surgical intervention, avoiding the delays and potential hazards associated with custom-made implants.

The 72% implant survival rate observed in this series corresponds closely to previously reported results from larger multicenter cohorts, in which implant survival ranged from 65% to 89% at mid-term follow-up [28]. It is also similar to the results reported by Rizkallah et al. [29]. However, the survival rate of a conventional total hip arthroplasty is higher, at 85.7% after 15 years [30]. The high revision rate in our study collective of 67% reflects the complexity of the underlying pathology and is not unexpected in this patient population but rather comparable to the literature with reference values of 75% [28]. Instability and infection were the dominant complications, consistent with the major causes of failure reported in the literature [28]. The proportion of early periprosthetic infections (≈17%) aligns with earlier data and highlights the persistent challenge of infection control in pelvic reconstructions.

In our series, the incidence of deep infection was 16.7%, which is consistent with the lower range reported in previous studies of pelvic megaprostheses [24,31]. Similar to earlier reports, most infections occurred early in the postoperative period and were frequently associated with instability as an initiating factor. In 80% of patients who developed instability as a postoperative complication, a prosthetic infection occurred subsequently as a secondary complication. A revision procedure is a known risk factor for prosthetic infection. In the study by Kosashvili et al., the probability of prosthetic infection increased with the number of revision procedures [32]. Poss et al. showed in their study that the risk of infection was increased by up to eightfold [33]. Reasons cited for the increased infection risk included dead space, scar tissue and longer operative time. The increased infection rates following instability may be attributable to revision surgeries, as the primary implantation of a hemipelvic prosthesis is already associated with an elevated risk of infection. However, early revision within 21 days did not seem to adversely affect implant survival, suggesting that the prompt surgical management of complications can preserve implant function and should not be delayed for fear of jeopardizing the reconstruction. Nonetheless, a higher implant survival was observed in patients who did not undergo revision surgery. However, given the small number of events and the resulting limited statistical power, this finding should be interpreted purely descriptively. This subgroup comparison is exploratory in nature, and any apparent differences may be subject to substantial uncertainty. The potential association between repeated surgical interventions and increased complication risk as previously suggested by Kosashvili et al. and Poss et al., ultimately leading to explantation of the prosthesis, therefore remains hypothetical in this cohort and requires confirmation in larger, adequately powered studies.

In our study, metastatic disease may reduce overall patient survival but did not seem to influence implant survival. The study by Tepper et al. demonstrated that implant survival exceeded patient survival (mean 10.4 months) in individuals undergoing hip arthroplasty for metastatic bone disease [34]. Similarly, Schneiderbauer et al. reported a median survival of 8.6 months and found that postoperative complications did not significantly affect overall survival [35]. This finding may support the concept that oncologic prognosis is primarily determined by tumor biology rather than the reconstruction method.

Studies have reported MSTS values ranging between 47% and 70% [8,28,29] after pelvic reconstruction with the LUMiC^®^ endoprosthesis. These outcomes confirm that even in a high-risk patient group with extensive bone loss, the LUMiC^®^ system allows satisfactory limb function and ambulatory capacity. Since only three patients completed the questionnaire in our study, functional results can only be assessed on an exploratory basis. The reported MSTS of 59% and TESS of 73% may suggest that limb function is comparable to that reported in the other studies. However, a larger cohort is required to confirm this hypothesis. Possible reasons for the low number of completed questionnaires include the long observation period of the study. Over the nearly 15-year follow-up period, some patients may have changed their contact information. It is also possible that willingness and interest in participating in the study declined over time.

This study has several limitations inherent to its retrospective, single-center design. The small sample size and incomplete follow-up restrict statistical power. With respect to oncological treatment, no data are available regarding neoadjuvant or adjuvant therapies. Such treatments may significantly influence wound healing, infection risk, and overall survival. The lack of this information therefore represents a relevant limitation of this study. Although only 18 patients were included in our study, the LUMiC^®^ prosthesis is rarely used in tumor endoprosthetic surgery. Guzik reported on six patients treated between 2011 and 2015 [36]. Likewise, the study by Klopper et al. [37] included only 13 patients, further emphasizing the rarity of the indication for the implantation of the LUMiC prosthesis. The patient population was heterogeneous regarding diagnosis and extent of resection. Both the small size and the heterogeneity of the cohort represent major limitations of the study. Functional assessment was available for only three patients and must be interpreted descriptively. Nevertheless, the long observation period of over a decade and the consistent surgical technique provide meaningful insight into real-world outcomes of this complex reconstruction. In contrast to previous LUMiC^®^ studies, the present series contributes extended follow-up data and includes both oncologic and non-oncologic cases, as well as detailed information on the implants used. In addition, the detailed analysis of revision suggests a possible association between the type of initial complication and the subsequent development of prosthetic infection. Furthermore, these data align with study collectives known from the literature with similar patient numbers.

## 5. Conclusions

The LUMiC^®^ modular endoprosthesis can be used for the reconstruction of periacetabular bone defects after tumor resection or failed arthroplasty. Hemipelvic prostheses are associated with an increased complication rate at the time of primary implantation, which in turn is linked to an increased risk of subsequent complications during revision procedures. This elevated complication burden may contribute to the reduced survival rates observed compared with conventional total hip arthroplasty. Nevertheless, implant survival in the present series was comparable to that reported in other studies investigating the reconstruction of periacetabular defects. In our study, early revision does not seem to compromise implant survival. Metastatic bone disease appeared to limit patient survival but did not have a significant impact on implant survival. However, the retrospective design of the study as well as the small and heterogenous cohort are important limitations that may affect the interpretation of the results. Furthermore, the reported TESS and MSTS scores should be considered exploratory given the very limited number of evaluable patients. The ongoing optimization of surgical technique and infection prevention remains essential.

## Figures and Tables

**Table 1:** Descriptive analysis of demographic parameters and indication for surgery (*n* = 18).

Age	Mean	Standard Deviation
	58.92	19.23
**Sex**	**Number**	**Percentage in %**
Male	8	44.44
Female	10	55.56
**Indication for surgery**		
Chondrosarcoma	6	33.33
Osteosarcoma	2	11.11
Pathological acetabular fracture due to metastasis	3	16.67
Pathological acetabular fracture due to synovial sarcoma	1	5.55
Ewing’s sarcoma	1	5.55
Myoepithelial carcinoma	1	5.55
Malignant peripheral nerve sheath tumor	1	5.55
Solitary fibrous tumor	1	5.55
Periprosthetic fracture	1	5.55
Prosthetic failure	1	5.55

**Table 2:** Descriptive analysis of tumor histology (*n* = 16).

	Number	Percentage in %
Patient without tumor diagnosis	2	11.11
**TNM-classification**		
Tx	2/16	12.50
T1	3/16	18.75
T2	6/16	37.50
T3	4/16	25.00
T4	1/16	6.25
N1	0	0.00
Nx	2/16	12.50
M1	5/16	31.25
Disseminated metastasis	3/5	60.00
Disseminated metastasis (postoperative)	1/5	20.00
Singular metastasis	1/5	20.00
**Grading**		
Gx	3/16	18.75
G1	1/16	6.25
G2	5/16	31.25
G3	6/16	37.50
G4	1/16	6.25

**Table 3:** Descriptive analysis of surgical treatment (*n* = 18).

Level of Resection (Enneking Classification)	Number	Percentage in %
No oncological resection	4	22.22
P1 + 2	2	11.11
P2	5	27.78
P2 + 3	7	38.89
**Site of surgery**		
Right	9	50.00
Left	9	50.00
**Patients without previous surgery at the same site**	13	72.22
**Patients with previous surgery at the same site**	4	22.22
Hip arthroplasty	2/4	50.00
Curettage and cement plombage	¼	25.00
Segment resection	¼	25.00
	**Mean**	**Standard Deviation**
Duration of surgery in minutes	441.2	194.50
Postoperative length of hospital stay in days	38.78	25.71

**Table 4:** Descriptive analysis of used LUMiC^®^ implants (*n* = 18).

Cup Size	Number	Percentage in %
50	3	16.67
54	9	50.00
60	6	33.33
Cemented cup	0	0.00
**LUMiC stem size**		
Unknown	1	5.55
8 × 65	2	11.11
8 × 75	1	5.55
8 × 85	10	55.56
10 × 75	2	11.11
10 × 85	2	11.11
**Femoral stem size**		
MUTARS 14 × 120	3	16.67
MUTARS RS 14 × 150	1	5.55
MUTARS RS 16 × 250	1	5.55
MUTARS RS 18 × 200	1	5.55
Fitmore B 5	1	5.55
Ecofit 10	2	11.11
Ecofit 11.25	1	5.55
Ecofit 12.5	1	5.55
Ecofit 13.75	1	5.55
Ecofit 15	1	5.55
Ecofit 17.5	3	16.67
Unknown	1	5.55
No stem replacement	1	5.55
Cemented femoral stem	0	0.00
**Dual-mobility cup**	10	55.56

**Table 5:** Descriptive analysis of complications and revision (*n* = 18).

First Occurring Complication (Henderson Classification)	Number	Percentage in %
No complication	7	38.89
1A (instability)	5	27.78
1B (aseptic wound dehiscence)	1	5.55
2A (aseptic loosening < 2 years after implantation)	0	0.00
2B (aseptic loosening > 2 years after implantation)	1	5.55
3A (prosthetic failure)	0	0.00
3B (periprosthetic fracture)	1	5.56
4A (periprosthetic infection < 2 years after implantation)	3	16.67
4B (periprosthetic infection > 2 years after implantation)	0	0.00
**Other complications**		
R1 resection	1	5.55
Lymphocele	1	5.55
**Secondary complication occurring subsequent to complication 1A**		
4A	4/5	80.00
**Secondary complication occurring subsequent to complication 3B**		
4A	1/1	100.00
**Secondary complication occurring subsequent to complication 4A**		
1A	1/3	33.34
**Patients undergoing at least one revision surgery**	12	66.67
	**Mean**	**Standard Deviation**
Days until first revision (1 outlier)	21.55	12.84
Number of revisions per revision patient	3.16	2.55

**Table 6:** Descriptive analysis of questionnaires (*n* = 3).

Questionnaires	Number	Mean	Standard Deviation
MSTS	3/18	58.89	21.43
TESS	3/18	73.37	6.836

**Figure A1:**
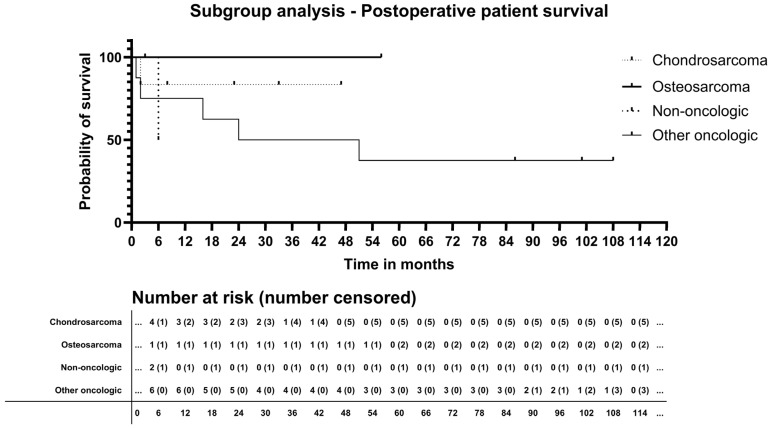
Kaplan–Meier analysis of overall survival following LUMiC^®^ prosthesis implantation. Subgroup analysis for patients diagnosed with chondrosarcoma, osteosarcoma, other oncological diagnoses and non-oncological diagnoses is shown. The difference between groups was evaluated using the log-rank test (*p* = 0.5225). Survival probability is shown over time in months. Tick marks indicate censored observations. The number of patients at risk is displayed below the *x*-axis. The event was defined as death from any cause. Patients without an event at last follow-up were censored at that time point.

**Figure A2:**
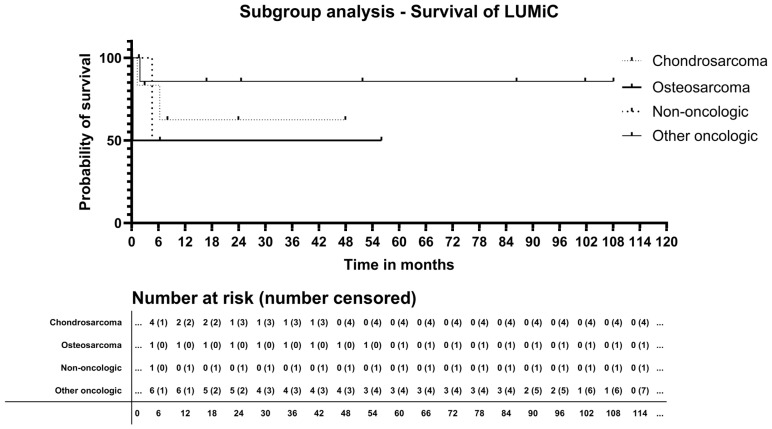
Kaplan–Meier curve survival analysis for LUMiC^®^ prosthesis. Subgroup analysis for patients diagnosed with chondrosarcoma, osteosarcoma, other oncological diagnoses and non-oncological diagnoses is shown. The difference between groups was evaluated using the log-rank test (*p* = 0.5663). Survival probability is shown over time in months. Tick marks indicate censored observations. The number of patients at risk is displayed below the *x*-axis. The event was defined as complete explantation of the LUMiC^®^ prosthesis. Patients without an event at last follow-up were censored at that time point.

## Data Availability

The original contributions presented in this study are included in the article. Further inquiries can be directed to the corresponding author.

## Abbreviations

The following abbreviations are used in this manuscript:

MSTS	Musculoskeletal Tumor Society Score
TESS	Toronto Extremity Salvage Score
SD	Standard deviation
